# Contribution of Statins towards Periodontal Treatment: A Review

**DOI:** 10.1155/2019/6367402

**Published:** 2019-02-27

**Authors:** Catherine Petit, Fareeha Batool, Isaac Maximiliano Bugueno, Pascale Schwinté, Nadia Benkirane-Jessel, Olivier Huck

**Affiliations:** ^1^INSERM (French National Institute of Health and Medical Research), UMR 1260, Regenerative Nanomedicine, Fédération de Médecine Translationnelle de Strasbourg (FMTS), Strasbourg, France; ^2^Université de Strasbourg, Faculté de Chirurgie-dentaire, 8 rue Sainte-Elisabeth, 67000 Strasbourg, France; ^3^Hôpitaux Universitaires de Strasbourg, Pôle de Médecine et Chirurgie Bucco-dentaire, Department of periodontology, 1 place de l'Hôpital, 67000 Strasbourg, France

## Abstract

The pleiotropic effects of statins have been evaluated to assess their potential benefit in the treatment of various inflammatory and immune-mediated diseases including periodontitis. Herein, the adjunctive use of statins in periodontal therapy *in vitro*, *in vivo*, and in clinical trials was reviewed. Statins act through several pathways to modulate inflammation, immune response, bone metabolism, and bacterial clearance. They control periodontal inflammation through inhibition of proinflammatory cytokines and promotion of anti-inflammatory and/or proresolution molecule release, mainly, through the ERK, MAPK, PI3-Akt, and NF-*κ*B pathways. Moreover, they are able to modulate the host response activated by bacterial challenge, to prevent inflammation-mediated bone resorption and to promote bone formation. Furthermore, they reduce bacterial growth, disrupt bacterial membrane stability, and increase bacterial clearance, thus averting the exacerbation of infection. Local statin delivery as adjunct to both nonsurgical and surgical periodontal therapies results in better periodontal treatment outcomes compared to systemic delivery. Moreover, combination of statin therapy with other regenerative agents improves periodontal healing response. Therefore, statins could be proposed as a potential adjuvant to periodontal therapy. However, optimization of the combination of their dose, type, and carrier could be instrumental in achieving the best treatment response.

## 1. Introduction

Periodontitis is an inflammatory disease of infectious origin characterized by progressive destruction of periodontal soft and hard tissues leading to tooth loss. The main symptoms comprise gingival inflammation, formation of periodontal pocket, alveolar bone loss, abscess, or tooth mobility [1]. The pathogenesis of periodontitis involves a complex interaction of immune and inflammatory cascades initiated by bacteria of the oral biofilm [2]. Persistent inflammation and dysbiosis worsen periodontal tissue damage, and the host response plays a vital role in this phenomenon contributing to tissue destruction [3].

The conventional treatment comprising scaling and root planing (SRP) presents limitations in certain cases involving deep periodontal pockets, inaccessible areas, or severe periodontitis [4]. Therefore, several adjunctive pharmacological therapeutics have been tested to improve its outcomes. In this context, systemic and local deliveries of drugs such as antibiotics, bisphosphonates, anti-inflammatory drugs, anticytokines, probiotics, and prebiotics have been tested so far to reduce bacterial load and to control inflammation [5–9]. Likewise, the use of statins in periodontal treatment has been explored recently [10]. Statins, or inhibitors of 3-hydroxy-3-methylglutaryl coenzyme A reductase (HMG-CoA reductase), are a group of drugs, used primarily to treat hyperlipidemia and to prevent cardiovascular diseases [11]. After their discovery in the 70s, they have been widely prescribed worldwide [12]. They differ mainly in their ring structure, and these structural differences modify their pharmacological properties including hydrophilicity and lipophilicity. The lactone ring is present in an active form (already hydrolyzed) in all statins except for simvastatin, lovastatin, and mevastatin, in which the lactone ring is activated (hydrolyzed) in the liver. The lactone form of the statins enables their transport, metabolism, and clearance [13] (Table 1).

Apart from their lipid-lowering properties, statins possess pleiotropic effects due to their anti-inflammatory, antioxidative, antibacterial, and immunomodulatory properties [14–17]. Statins have also been reported to have anabolic effects on the bone by augmenting bone morphogenetic protein-2 (BMP-2) expression, thus contributing towards the differentiation and activity of osteoblasts (OBs) [18]. In view of their beneficial properties, statins have been presented as new potential candidates for improving periodontal therapy outcomes [19, 20].

In several preclinical and clinical studies, statins have exhibited contradictory results [21–23] depending on the mode of delivery (local vs systemic), anatomy and severity of the lesions, type of disease, and treatment approach (nonsurgical vs surgical). Therefore, the aim of this literature review was to establish a better understanding of the prophylactic and therapeutic effects of all statin types administered locally or systemically as adjuvant to nonsurgical/surgical periodontal treatment in existing preclinical models and clinical settings and to explore the biological mechanisms underlying these healing and proregenerative effects in the management of periodontitis.

## 2. Methods

### 2.1. Literature Search

Studies published in English language only were included, and the last search was carried out in September 2018. Regarding studies performed on animal models and clinical trials, a systematic literature search was performed in the PubMed/MEDLINE and ScienceDirect databases. A hand search has also been performed after checking references of the identified articles. Concerning *in vivo* studies, the following keywords were used for the search: periodontitis OR periodontal disease OR alveolar bone loss OR periodontal attachment loss OR periodontal pocket AND simvastatin OR statin OR rosuvastatin OR atorvastatin OR cerivastatin OR mevastatin OR lovastatin OR pravastatin OR Fluvastatin OR pitavastatin OR Hydroxymethylglutaryl-CoA Reductase Inhibitors AND mouse OR dog OR pig OR rat OR rodent OR rabbit OR monkey OR in vivo. A study was considered eligible if it met the following criteria: (1) experimentally induced periodontitis (EIP) and/or acute/chronic periodontal defects (ACP), (2) treatment of EIP and/or ACP with statins (local or systemic or combination) with or without SRP or other periodontal treatment modalities, and (3) at least one periodontal parameter assessed as outcome. Exclusion criteria for *in vivo* studies were the following: (1) periapical lesions, (2) tooth extraction models, (3) orthodontic movements, (4) calvarial models, (5) long bone defects, and (6) drug-induced gingival enlargement.

Concerning clinical studies, the following keywords were used for the search: periodontitis OR periodontal disease OR alveolar bone loss OR periodontal attachment loss OR periodontal pocket AND simvastatin OR statin OR rosuvastatin OR atorvastatin OR cerivastatin OR mevastatin OR lovastatin OR pravastatin OR Fluvastatin OR pitavastatin OR Hydroxymethylglutaryl-CoA Reductase Inhibitors. A study was considered eligible if it met the following criteria: (1) randomized and controlled clinical trials, (2) cohort clinical studies, (3) longitudinal studies, (4) patients with diagnosis of chronic or aggressive periodontitis, (5) systemic or local administration of statins with nonsurgical or surgical periodontal treatment, and (6) at least one periodontal parameter: pocket depth (PD), clinical attachment level (CAL), bone loss (BL), or tooth loss (TL) assessed as outcome. Exclusion criteria for clinical studies were the following: (1) no follow-up, (2) no periodontal treatment, and (3) reviews, letters, and case reports.

### 2.2. Study Selection

Titles and abstracts of the studies were screened independently by two reviewers (CP and FB) and categorized as suitable or not for inclusion. Full reports were reviewed independently for studies appearing to meet the inclusion criteria or for which there was insufficient information in the title and abstract to allow a clear decision. Disagreements between the authors were resolved after discussion with a third reviewer (OH).

### 2.3. Risk of Bias Assessment

Risk of bias was assessed using the Cochrane Collaboration's tool for assessing risk of bias which provided guidelines for the following parameters: sequence generation, allocation concealment method, blinding of the examiner, address of incomplete outcome data, and free of selective outcome reporting. The degree of bias was categorized as follows: low risk if all the criteria were met, moderate risk when only one criterion was missing, and high risk if two or more criteria were missing. Two reviewers (FB and CP) independently performed the quality assessment, and any disagreement was resolved by a third investigator (OH) (Supplemental Table 1).

## 3. Results

### 3.1. Effect of Statins on the Inflammatory-Immune Crosstalk

Localization of *periodontium* at the interface between the teeth and jaws exposes periodontal tissues to continuous bacterial challenge which could contribute to exacerbation of the immune response during periodontal wound healing. Recruitment of inflammatory cells at the periodontal site, including polymorphonuclear (PMN) leukocytes, macrophages, and lymphocytes, is associated to the release of a complex nexus of cytokines. When the inflammatory front migrates toward the alveolar bone, it stimulates osteoclastogenesis and subsequent alveolar bone destruction [24]. Therefore, the importance of inflammation control at the soft tissue level cannot be undermined.

**Figure 1:**
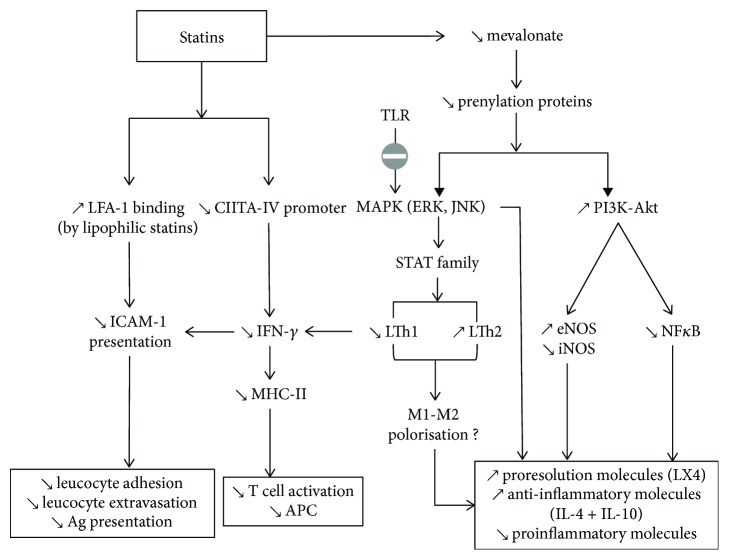
Effect of statins on the inflammatory-immune crosstalk. Direct LFA1 site binding by lipophilic statins decreases ICAM-1 presentation leading to reduced leukocyte chemotaxis and antigen presentation. Statins inhibit MHC-II induction by IFN-*γ* leading to decreased T-cell activation. Statins lower mevalonate release, leading to resolution of inflammation via the ERK, MAPK, and PI3K-Akt pathways.

The effects of statins on the inflammatory-immune crosstalk involved in the periodontal wound healing have been evaluated. Statins decrease the levels of proinflammatory cytokines (interleukin-1 beta (IL-1*β*), interleukin-8 (IL-8), interleukin-6 (IL-6), and tumor necrosis factor-alpha (TNF-*α*)) and increase the release of anti-inflammatory mediators (IL-10) and chemokines [25, 26]. There are several pathways implicated in the action of statins, notably suppression of HMG-CoA reductase, thereby inhibiting Rac and p21Ras phosphorylation. As Rac and p21Ras are coupled to the transcription of proinflammatory molecules via MAP kinase (MAPK) pathways, therefore, statins also suppress nuclear factor kappa B (NF-*κ*B) activation, thus reducing the expression of proinflammatory molecules [27] (Figure 1).

#### 3.1.1. Effect of Statins on Inflammatory Molecules

*In vitro*, the effect of statins on inflammatory mediators' secretion was demonstrated to be cell specific. For instance, in human oral epithelial cells [15] and OBs [28], statins reduced IL-6, IL-8 release, whereas, in T-cells [29, 30], statins increased the expression of IL-4, IL-5, IL-10 and IL-13. *In vivo*, statins confirmed the reduction of cyclooxygenase-2 (COX-2), prostaglandin E_2_ (PGE_2_), IL-1*β*, IL-6, IL-8, TNF-*α*, interferon-gamma (IFN-*γ*), C-reactive protein (CRP), colony-stimulating factors (CSF2, CSF3), recruitment of mononuclear inflammatory cells, and several Toll-like receptors (TLRs) in various EIP or ACP models [26, 31–35]. Clinical trials also corroborated the downregulation of inflammation by the use of statins, as demonstrated by increased IL-10 level in gingival crevicular fluid (GCF) from hyperlipidemic patients treated with statins [19].

#### 3.1.2. Effect of Statins on Proresolution Molecules

Periodontal wound healing and regeneration involve a constant “tug-of-war” between the proinflammatory and anti-inflammatory/proresolution mediators [36, 37]. Anti-inflammatory effects of statins enhancing resolution of periodontal inflammation, that is, initiated by several endogenous chemical and lipid mediators, such as the lipoxins (LXs), resolvins (RVs), protectins, and maresins, could possibly explain the positive treatment outcomes [38, 39]. However, further studies need to explore the exact effect of statins on the proresolution mediators.

#### 3.1.3. Effect of Statins on Host Modulation

Literature reports contradictory results regarding the effect of statins on different types of immune cells. For instance, in an ACP model, simvastatin did not change circulating white blood cell (WBC) counts in a study [33], whereas leukocyte infiltration was decreased by atorvastatin gavage in an EIP model [40]. Similarly, regulatory T (Treg) cells that control adaptive immunity against pathogens and activate other effector immune cells were reported to be regulated by statins. In this regard, atorvastatin and simvastatin demonstrated an increase in the number of human Treg cells and differentiation of CD4 into Treg *in vitro* [41, 42].

Furthermore, TLRs have an important role in the immune-inflammatory crosstalk with a consequent impact on periodontal wound healing response. In the context of periodontal treatment, targeting TLRs has been proposed as it could enhance antimicrobial properties, suppress adverse inflammation, or activate tissue repair [43]. Interestingly, simvastatin inhibited the stimulation of several TLRs (1, 2, 3, 4, 6, 7, and 9) by *Aggregatibacter actinomycetemcomitans* (*A.a*) LPS *in vivo*, reducing its capability to escape innate immune response [33]. Hence, statins play an instrumental role in the modulation of inflammatory and immune responses.

#### 3.1.4. Inhibition of Major Histocompatibility Complex Class II (MHC-II) by Statins

In case of nonresolving periodontal lesions, bacterial antigens are processed and presented by antigen-presenting cells and macrophages. Such process is associated to massive immune cell recruitment implicated in tissular destruction [2]. In this regard, statins are able to inhibit MHC-II expression due to inhibition of the inducible promoter IV of the class II transactivator (CIITA) as observed in several cell types, including monocytes and macrophages [44]. This effect renders statins to have a potential host-modulating impact on periodontal treatment.

#### 3.1.5. Lymphocyte Function-Associated Antigen-1 LFA1 Site Binding by Statins

Lymphocyte function-associated antigen-1 (LFA-1), an integrin with its main ligand intercellular adhesion molecule-1 (ICAM-1), is activated on the surface of fibroblasts (FBs) by IFN-*γ* and represents a critical phase in the early stage of inflammation. ICAM-1 regulates LFA-1-dependent neutrophil transmigration and recruitment to the inflammation site [45]. Several studies have demonstrated the inhibition of LFA-1 by statins in many inflammatory and immune diseases other than periodontitis. Statins inhibit ICAM-1 upregulation and chemotaxis of monocytes [46]. Lovastatin, simvastatin, and mevastatin, but not pravastatin, were able to inhibit the LFA-1/ICAM-1 interaction *in vitro* by binding to the L-site of LFA-1 [47]. In this way, statins limit the exacerbation of immune-mediated inflammatory response at the lesion site. However, the impact of statins on LFA-1 binding in the context of periodontal wound healing remains unexplored.

#### 3.1.6. Effect of Statins on Nitric Oxide Synthase (NOS)

NOS plays an important role in host defence and homeostasis and has been implicated in the pathogenesis of periodontitis, where it is expressed in FBs, epithelial cells, rests of Malassez, macrophages, osteoclasts (OC), and vascular endothelial cells [48, 49]. In chronic periodontitis, bacterial challenge induces proinflammatory cytokine release and a higher expression of inducible NOS (iNOS) and NOS derived from FBs and WBCs that migrate to the periodontal lesion [50–52] leading to inflammation-mediated bone resorption [53]. Various studies demonstrated a NOS-inhibiting effect by the use of statins. For instance, *in vivo*, rosuvastatin significantly reduced inflammation-mediated tissue destruction and gingival iNOS expression [54].

Concerning the underlying mechanism of action, statins attenuate the production of reactive oxygen species (ROS) induced by NADPH oxidase by suppressing Rac's geranylation. Phosphatidylinositol-3 active kinase (PI3-Akt) is a kinase that phosphorylates and stimulates eNOS. Mevalonate is able to inhibit PI3-Akt; therefore, by reducing the concentration of mevalonate, statins upregulate eNOS-derived NO production resulting in vasorelaxation that leads to improved angiogenesis and wound healing response [27].

#### 3.1.7. Effect of Statins on Matrix Metalloproteinases (MMPs)

MMPs degrade extracellular matrix proteins, especially collagen, contributing to the degradation of periodontal tissue including alveolar bone [55]. Most statins have been reported to potently inhibit the expression of MMP-1, MMP-8, and MMP-9 upregulated by LPS as demonstrated for simvastatin in mononuclear cells *in vitro* [56]. Moreover, *in vivo*, a decrease of MMP-1, MMP-2, MMP-8, and MMP-9 was observed by the use of statins [31, 57–59]. Thus, statins prevent periodontal tissue and alveolar bone destruction by inhibiting the release of MMPs.

### 3.2. Effect of Statins on Bone Metabolism

**Figure 2:**
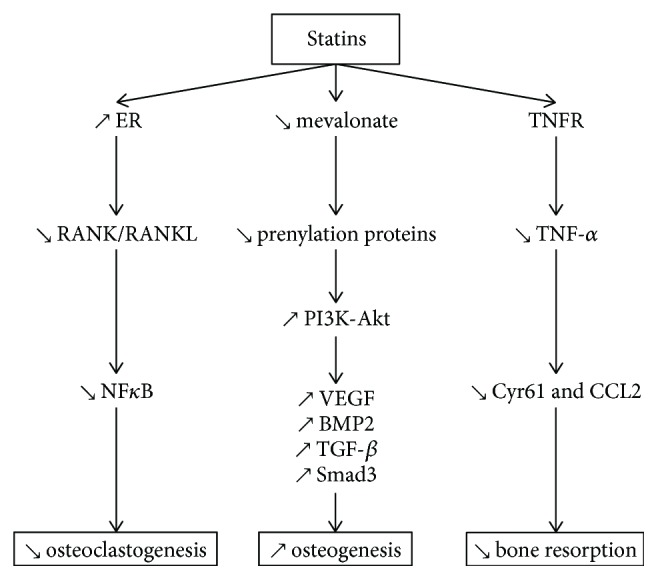
Effects of statins on several pathways involved in bone metabolism. Statins decrease osteoclastogenesis via RANK/RANKL and NF-*κ*B signaling. Statins promote osteogenesis by increasing VEGF, BMP2, and TGF-*β* expression through the PI3-Akt pathway. Statins prevent inflammation-mediated bone resorption by decreasing TNF-*α*, via TNFR.

Statins have an impact on bone metabolism through increase of osteogenesis, decrease of OB apoptosis, and osteoclastogenesis [60]. Statins allow periodontal regeneration via the Ras/Smad/extracellular signal-regulated kinase (Erk)/BMP-2 pathway that enhances bone formation [61] and by antagonizing TNF-*α* through Ras/Rho/mitogen-activated protein kinase (MAPK) that causes osteoclastic differentiation [62]. Moreover, they significantly increase OB differentiation factors such as alkaline phosphatase (ALP), osteocalcin (OCN), bone sialoprotein (BSP), BMP-2 [63], osteopontin (OPN), and vascular endothelial growth factor (VEGF) [64] (Figure 2).

#### 3.2.1. Role of Statins in the Promotion of Osteogenesis

Inhibition of HMG-CoA by statins decreases prenylation of farnesyl pyrophosphate (FPP) and geranylgeranyl pyrophosphate (GPP) leading to increased levels of BMP-2 and VEGF through the PI3-Akt pathway. Interestingly, both VEGF and BMP-2 regulate OB differentiation and bone formation during bone repair and regeneration [65, 66]. Concerning BMP, simvastatin and lovastatin increased the levels of BMP-2, consequently, increasing OB activity *in vitro* [58, 63]. Statins present a cost-effective option when compared with growth factors such as BMP-2 [67, 68].

Hydrophobic statins (simvastatin, atorvastatin, and cerivastatin) also increased mRNA expression of VEGF in OBs [69]. Likewise, simvastatin increased osteoprotegerin (OPG) expression in periodontal tissue [58] and enhanced matrix calcification in human bone marrow stem cells by diminishing the mean size of the fibroblastic colony-forming units (CFU-Fs) [70]. *In vivo*, statins stimulated bone growth and repair by increasing angiogenesis [71]. In particular, the lactone-form statins (lovastatin and simvastatin) stimulated OB differentiation of mouse periodontal ligament cells (PDLs) via the ERK1/2 pathway (phosphorylation) and enhanced intercellular matrix mineralization [63].

#### 3.2.2. Role of Statins in the Inhibition of Bone Destruction

Statins act through certain pathways that avert bone degradation. Several clinical trials confirm the reduction of alveolar bone loss by statins, as an adjunct to SRP [72]. Many studies reported significantly decreased bone resorption by the use of simvastatin, rosuvastatin, and atorvastatin [26, 28, 32, 73]. Interestingly, simvastatin reduced TNF-*α*-induced synthesis of Cysteine-rich 61 (Cyr61) and chemokine ligand 2 (CCL2) [74] that are potential osteolytic mediators in inflammatory bone diseases, in human OB, thereby decreasing bone loss. Besides, statins increase bone formation by inhibiting OB apoptosis, augmenting TGF-*β* against the Smad3 signaling pathway. As an evidence, pitavastatin, mevastatin, and simvastatin induced the expression of Smad3 in nontransformed OBs (MC3T3-E1) [75]. Consequently, statins prevent bone destruction and also promote bone healing and regeneration.

#### 3.2.3. Role of Statins in the Inhibition of Osteoclastogenesis

Statins suppress osteoclastogenesis through the OPG/receptor activator of the nuclear factor kappa-B ligand (RANKL)/RANK signaling pathway. Statins (simvastatin, atorvastatin, and fluvastatin) inhibited, *in vitro* and *in vivo*, the expression of the receptor activator of RANK which along with RANKL is required for the differentiation of OC precursors [26, 31, 33, 58, 76]. Nevertheless, IL-10 is also implicated in inhibiting bone resorption by preventing the RANK/RANKL pathway ([77]); hence, statins could potentially reduce the inflammation-mediated bone resorption [25]. Another mechanism for osteoclastogenesis involving unprenylated Rap GTP-binding protein 1A (Rap-1A), a RAS super family of small GTP-binding protein member, has been studied in the context of statins. Rosuvastatin, pravastatin, cerivastatin, and simvastatin caused accumulation of unprenylated Rap-1A in rabbit osteoclast-like cells and macrophages, inhibiting osteoclast-mediated resorption. Interestingly, hydrophilic statin (cerivastatin) was more effective than hydrophobic statin (rosuvastatin) to inhibit OC prenylation [78]. Additionally, the mRNA expression of cathepsin K, a key marker of OC differentiation, is reduced by simvastatin through inhibition of Src signaling and modulation of MAPK including ERK1/ERK2. Moreover, upregulation of AKT leads to a decrease of OC activity via RANKL and BMP-2 [79].

### 3.3. Antibacterial Effect of Statins

**Figure 3:**
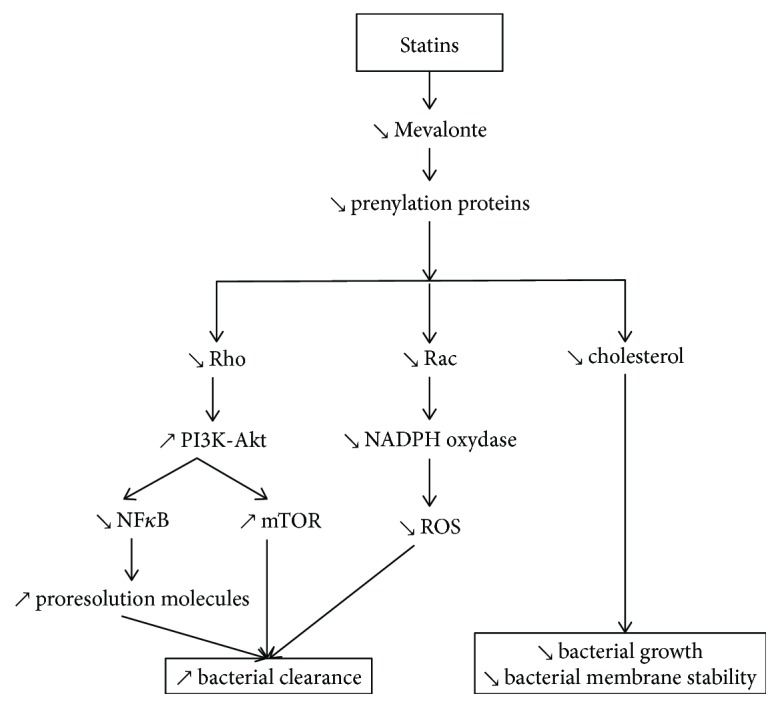
Antibacterial effect of statins. Statins arrest bacterial growth and disrupt their membrane stability by decreasing cholesterol. Statins increase bacterial clearance by decreasing NF-*κ*B and ROS signaling (via the PI3K-Akt and NADPH oxidase pathways, respectively) and by enhancing proresolution molecule release.

Periodontitis is a polymicrobial disease involving keystone pathogen such as *Porphyromonas gingivalis* (*P.g*) that is able to hijack the adaptive immune response. Therefore, elimination of the periodontal pathogens is the cornerstone of periodontal treatment. Uncontrolled infection hinders periodontal wound healing and may worsen the therapeutic outcome by reducing the clinical attachment gain. Statins exhibit antimicrobial effects attributed to an increased bacterial clearance from the infection site as demonstrated in a model of sepsis (Figure 3) ([80]). Hence, statins could provide an additional benefit during periodontal wound healing (Table 2).

Cholesterol is an integral component needed by bacteria for maintaining their membrane integrity. Statins can counter bacteria by inhibiting the intermediate in the isoprenoid biosynthesis pathway necessary for membrane stability, which is substituted by cholesterol and protects bacteria from the toxic effect of statins. Statins, therefore, kill bacteria directly and by lowering accessible host cholesterol content for bacterial growth and protection. Such effects may be due to the disruption of teichoic acid structures reducing biofilm formation ([81]). Statins display antibacterial activity towards anaerobic bacteria, including periodontal pathogens such as *A.a* and *P.g*. For instance, low concentration of simvastatin was proven to be effective against *A.a* and *P.g* even if *A.a* was more sensitive (MIC < 1 *μ*g/mL) than *P.g* (MIC until 2 *μ*g/mL dilution) [82]. The hydrophobic nature of simvastatin may explain its antibacterial activity against periodontal pathogens where it disrupts the bacterial membrane in a “soap-like” manner causing its death [83]. Nevertheless, not all statins exhibit antibacterial activity. The degree of HMG-CoA reductase inhibition corresponds directly to the cholesterol-lowering capabilities of statins [84] but it does not seem commensurate with their antibacterial potency [85].

Some other mechanisms are modulated by the action of statins on lipoxin A4 (LXA4) production, a proresolving lipid mediator that enhances bacterial clearance, consequently reducing the severity of periodontal disease [86, 87]. Furthermore, the mechanistic target of rapamycin (mTOR) signaling, regulated principally by TLRs via two major pathways (NF-*κ*B-dependent pathway and a PI3-Akt-dependent pathway), is also involved in bacterial clearance [88]. It is known that statins inhibit isoprenoid synthesis, impeding intracellular signaling molecules like Rho or Rac [89].

Therefore, it is plausible that statins possess certain antibacterial properties that could facilitate periodontal treatment. However, since periodontitis is a polymicrobial disease, the susceptibility of various other periodontal pathogens to statins must also be evaluated.

### 3.4. Effects of Statins in Induced Periodontitis Models

**Figure 4:**
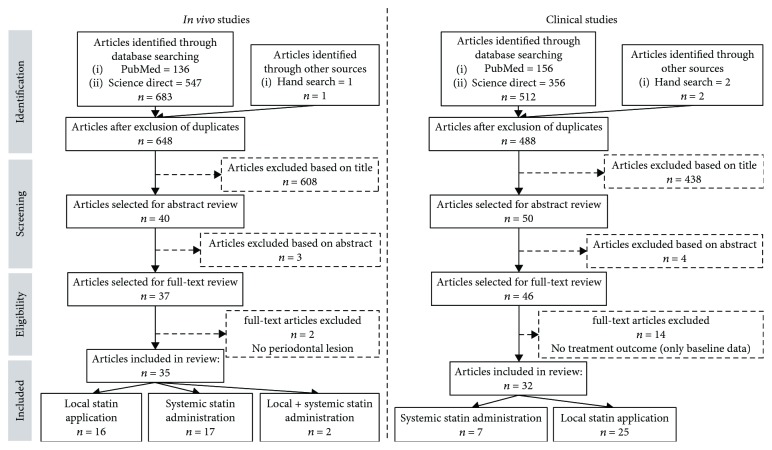
Selection of the studies.

Statins have been tested in several induced periodontitis models to evaluate improvement in periodontal parameters and their underlying biological mechanisms. *In vivo*, 35 studies were identified based on the inclusion criteria (Figure 4), out of which 16 involved local statin delivery (Table 3), 17 used systemic route (Table 4), and 2 employed a combination of both modes (Table 5). In the studies evaluating local statin application, 8 studies involved the treatment of EIPs while the remaining 8 investigated the treatment of ACP models, one of which was induced by LPS injection of *Escherichia coli* (*E. coli*) [90]. Concerning the systemic administration of statins (Table 4), 14 out of the total 17 studies treated EIPs, whereas the 3 remaining studies involved ACP models by LPS injections of *A.a* [32, 33] and *P.g* into the gingiva [76].

Regarding the mode of periodontitis induction, in total, 24 out of 35 studies had EIP with ligatures (cotton, nylon, or silk), whereas 11 used ACP including the 4 studies where periodontitis was induced by bacterial LPS. Studies were mostly performed in rodents (Tables 3, 4, and 5). In ACP models, the surgically created lesions were mainly intrabony defects, fenestration defects, dehiscence defects, furcation class II defects, and 3-walled intrabony defects.

In 6 studies, animals with systemic diseases (i.e, osteoporosis [26, 91, 92], metabolic syndrome [32], cyclosporine A-associated alveolar bone loss [35], hyperlipidemia [54], or hypertension [93] were used to evaluate the effect of statins treatment. Overall, 22 studies involved treatment with simvastatin, 7 with atorvastatin, 3 with rosuvastatin, 2 with lovastatin, and only one with fluvastatin. Some studies investigated more than one type of statin. *In vivo*, the systemic dosage used ranged from 0.3 to 30 mg/kg with 20 mg/kg as the most commonly tested dose. The dose of locally delivered statins varied with the type of carrier/scaffold used (Table 3). Five studies demonstrated insignificant improvements [94–98]. Interestingly, 3 of them involved surgical treatment of ACP models by local statin application [94, 96, 98] and one study employed nonsurgical local statin therapy [95], whereas only one EIP was treated with systemic statin delivery [97]. One study even demonstrated a negative impact of statin use [99].

### 3.5. Clinical Outcomes

The selected studies evaluating the effect of statins in the context of periodontal treatment included 23 controlled and randomized clinical trials, 8 cohort studies, and 1 longitudinal study (Figure 4). Primary outcomes varied between improvement of clinical attachment level (CAL), reduction of pocket depth (PD), tooth loss, radiographic bone defect depth, periodontal inflamed surface area (PISA), and serum and/or GCF proinflammatory cytokines level. Most of the studies focused on the local administration (*n* = 25) of statins (Table 6), while 7 investigated the impact of systemic route (Table 7). Essentially, effects of statins have been evaluated as an adjunct to both nonsurgical and surgical treatments, mainly in the context of chronic periodontitis in healthy patients.

### 3.6. Statins as a Local Adjunct to Nonsurgical Periodontal Treatment

The effect of local delivery of statins as an adjunct to nonsurgical periodontal therapy (SRP) was studied in 20 clinical trials (Table 6). Atorvastatin and simvastatin have been the most commonly studied statins. Amongst the identified studies, 13 demonstrated a significant PD reduction, CAL gain, and IBD fill in healthy patients, 2 in well-controlled type II diabetes patients, and 3 in smokers. At contrary, in 2 studies, the test groups using atorvastatin or simvastatin did not show any significant differences when compared with the control [21, 100]. For instance, with simvastatin, the mean PD gain was 1.23 ± 0.57 mm for the control group versus 1.83 ± 0.07 mm for the test group (*p* = 0,112) and the mean CAL gain was 2.09 ± 0.08 mm for the control group versus 2.43 ± 0.01 mm for the test group (*p* = 0.889) after 45 days. Nevertheless, authors found a statistically significant reduction of PI, BOP, IL-6, and IL-8 levels [21].

Only 4 studies compared the outcomes obtained with more than one statin; however, contradictory results were observed. For instance, one study did not show any significant difference between atorvastatin and simvastatin [100], whereas better results were obtained with atorvastatin in another study [101]. Nevertheless, two studies highlighted greater efficacy with rosuvastatin in comparison with atorvastatin [20, 102].

Interestingly, studies that have investigated the effects of statin treatment on the biological markers from GCF showed that simvastatin administration reduced significantly IL-6, IL-8 and increased the anti-inflammatory IL-10 [21, 100, 103].

### 3.7. Statins as a Local Adjunct to Surgical Periodontal Treatment

Statins have also been inspected for their role in the surgical treatment outcomes. In all identified studies where statins (simvastatin, atorvastatin, and rosuvastatin) were locally administered concomitant to surgical approach (including the use of biomaterials or PRF), a significant reduction of PD, improvement of CAL, and bone defect fill was achieved in the test group in comparison to the control group [104–108] (Table 6). Amongst these studies, the mean difference of PD between the test and control groups ranged from 1.3 ± 0.21 mm to 2.51 ± 0.22 mm (*p* < 0.001). Thus, the mean difference of CAL between the test and control groups ranged from 1.16 ± 0.09 mm to 2.35 ± 0.08 (*p* < 0.001). Moreover, the mean difference of bone defect fill between the test and control groups ranged from 1.336 ± 0.714 to 3.08 ± 0.07 (*p* < 0.001).

### 3.8. Impact of Systemic Administration of Statins on Nonsurgical Periodontal Treatment Outcomes

The impact of systemic administration of statins on nonsurgical periodontal treatment outcomes was evaluated in a few studies (Table 7). From the 7 studies identified, 4 demonstrated significant improvements regarding reduction of PD, CAL gain, and/or tooth loss in comparison to the control group [56, 109–111]. At contrary, 3 other studies did not show any significant differences in periodontal outcomes between the statin-treated and control groups [112–114]. These discrepancies could be due to the very short follow-up of the abovementioned 3 studies (3 months) compared to the other ones (from 3 months to 7 years follow-up). Moreover, one of the studies did not compare the treatment group with a control group [110].

## 4. Discussion

**Figure 5:**
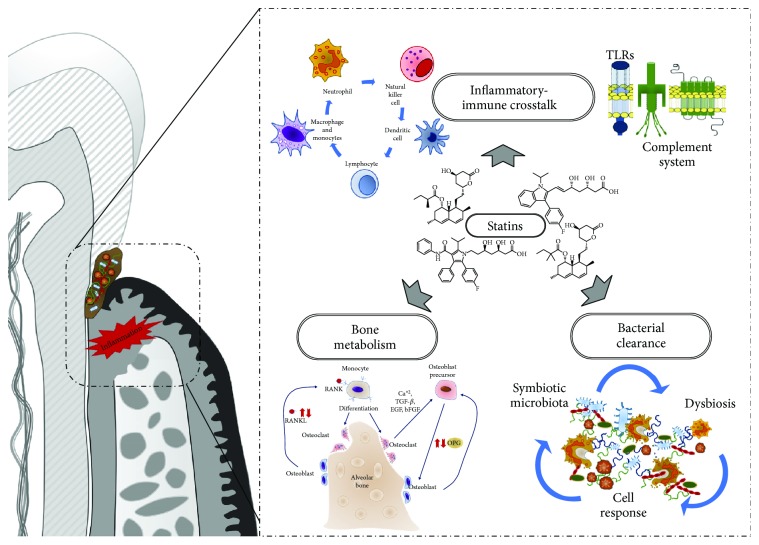
Pleiotropic effects of statins in the context of periodontitis management. Statin biological properties might be of interest for the management of periodontitis as they act on each tissular compartment and mechanisms including inflammatory-immune crosstalk, bone metabolism and bacterial clearance.

Statins exhibit multiple effects, including modulation of inflammatory-immune crosstalk, bone regeneration, and antibacterial activity, to promote periodontal wound healing and regeneration (Figure 5). They act through several closely interrelated pathways highlighting potential therapeutic targets. The hydrophobic or hydrophilic nature of statins determines their efficacy, action on periodontal pathogens, and treatment response and appears to be largely cell and tissue dependent [69, 78]. Further insight into this may help selecting the best statin.

Moreover, the mode of statin delivery also affects the treatment outcomes. Oral systemic administration of statins reduces periodontal inflammation and consequent tooth loss [111] but the low resultant dose available to the tissues after hepatic bypass renders them relatively less efficacious [60]. On the other hand, a higher dose to enhance efficacy can manifest systemic side effects such as statin-induced myopathy, hepatotoxicity, nephrotoxicity, pulmonary manifestations, ophthalmological manifestations, gastrointestinal hemorrhage risk, and oral manifestations (dryness, itch, bitterness, and cough) [115, 116]. Therefore, to avoid these side effects, various local application strategies have been tested that allow site-specific delivery reducing the required dose, frequency of application, and bioavailability in the blood [60, 117, 118], concomitantly improving patient compliance [119].

The development and selection of an optimal statin delivery carrier are crucial as it enhances the statin retention on the lesion and acts as a scaffold for cell growth and differentiation [120]; therefore, it should be capable to withstand the oral environment, continuous fluid exchange inside the pocket, and salivary influx.

Several studies demonstrate that anti-inflammatory properties of statins vary according to the type and dose of statin used [121]. On a cellular level, modulation of macrophage polarization from a proinflammatory M1 to a proresolution M2 phenotype by systemic delivery of immune modulatory drugs resolved persistent inflammation associated with chronic periodontitis [122]. In this context, statins' ability to switch M1 to M2 to promote periodontal wound healing and regeneration needs to be explored. Furthermore, it is yet to be established if statin-induced reduction in plasma total cholesterol and LDL cholesterol levels in the periodontal space could decrease macrophage recruitment to improve the treatment outcome.

Despite the documented anti-inflammatory properties of statins, a local high-dose statin application causes considerable soft tissue inflammation [123]. Accordingly, studies determined that reducing the simvastatin dose from 2.2 mg to 0.5 mg reduced inflammation without compromising its bone growth potential [67]. A 10 mg/kg/day dose in rats is equivalent to 70 mg/day for humans, so it is a high systemic dose compared to that commonly used in clinical practice (20-40 mg/day) [124].

Concerning locally applied statins, most clinical studies investigated the 1.2% dose (mainly atorvastatin, simvastatin, and rosuvastatin) [20, 23, 125, 126]. Therefore, other doses should be tested to compare efficacy.

Most of the review articles have focused on the use of statins as adjunct to the nonsurgical SRP in clinical settings [127–129]. Here, this review encompasses the use of statins (local, systemic, or combination), alone or in addition to other drugs or scaffolds, in nonsurgical or surgical periodontal treatment *in vitro*, *in vivo*, and in clinical trials. However, the potential of statins in surgical periodontal therapy remains relatively less explored except for a few studies where treatment outcomes were improved, primarily, with the combination of some other regenerative agents such as allograft or PRF [105, 106]. Cognizant of the numerous studies involving statins, not all statin types have been studied so far; thus, exploring all natural and synthetic statins to compare their efficacy and safety could be instrumental.

Notably, 17 out of 32 clinical studies were carried out by the same group of researchers on similar population; therefore, generalizations should be drawn with caution. Additionally, in most studies involving statins, the follow-up period was no longer than 9 months [103, 130]. Hence, it is imperative to follow clinical studies for periods longer than those commonly investigated so as to achieve a deeper and more genuine insight into their long-term benefits. Discrepancies amongst outcomes between time points are of importance to clearly conclude. For instance, the meta-analysis performed by Sinjab et al. [131] declared the outcomes of the control group of a study [20] to be better by considering the data up to 6 months follow-up, whereas the meta-analysis performed by Ambrósio et al. regarded the treatment group of the same study to have better outcomes as the follow-up data until 9 months was taken into account [132].

Moreover, the studies carried out so far mainly involved hyperlipidemic patients, diabetic patients, or smokers. Systemic diseases, such as obesity or metabolic syndrome, have been linked with periodontitis [133]. It has been demonstrated that such conditions modify significantly the host response to periodontal pathogens [134] but also could impaired treatment response. For instance, in a rat model of metabolic syndrome, the effects induced by statins in rats with metabolic syndrome were different in comparison with rats without [32] highlighting the potential modulation of pharmacologic effect due to the systemic condition. Even if clinical trials performed in diabetes patients or exhibiting hyperlipidemia showed promising results when statins were administered concomitantly to nonsurgical periodontal treatment [56, 110, 113, 114], more studies are required to better understand the differential biological mechanisms modulated by statin's administration. It would also be of importance to assess statins' tolerance and efficacy in subjects with different systemic conditions where periodontal treatment response is impaired (e.g., liver diseases, kidney dysfunction, and immunocompromised states).

In clinical trials, the local application of statins with surgical periodontal treatment always showed significant improvements in periodontal parameters [105, 106]. However, *in vivo*, statin application in ACP models showed contradictory results [99] which could be explained by the limitations of animal models to simulate conditions identical to human periodontal disease. Nevertheless, as a direct optimization of treatment protocols in humans is not ethically permissible, the utility of preclinical models to get directions and overall assessment of the expected treatment outcomes in clinical scenarios cannot be undermined.

Concerning the systemic administration of statins, a study reported that using a combination of two pharmacokinetically different statins (20 mg/day of atorvastatin plus 40 mg/day of pravastatin) in hyperlipidemic patients for one year improved their lipid profiles compared to those on monotherapies [135]. Besides, a case of a hyperlipidemic patient experiencing certain side effects with a high dose of systemic simvastatin who could well tolerate a combination of reduced doses of simvastatin and rosuvastatin instead has also been reported [136]. To the best of our knowledge, no two statins have been combined for periodontal treatment so far; nonetheless, combination of two statins could be tested for its impact on periodontal treatment response.

Likewise, the impact of incorporating statins with antimicrobial agents, growth factors, or other proregenerative molecules within a local application system could be studied as adjunct to SRP. Statin integration into gels [21] or dentifrice [137] could enhance ease of application and patient's compliance and could be potentially beneficial in the maintenance phase to counter periodontal breakdown that persists after conventional periodontal treatment. The literature does not report the impact of statins on patients with extremely poor oral hygiene; nonetheless, it could be interesting to explore the impact of statins on oral hygiene indicators.

## 5. Conclusion

Statins have been studied in depth in the context of bone regeneration, but soft tissue healing remains relatively less explored. Further research into it could present statins as a potential adjunctive therapeutic strategy with a positive impact on both hard and soft periodontal tissue healing. Furthermore, the impact of statins on proresolution molecules has not been investigated in the context of periodontal wound healing and regeneration. This could unveil new vistas for statins as regenerative therapeutics. Since all available statins have not been tested yet, new studies need to evaluate the impact of other statins on antibacterial, inflammatory, immune, and osteoprogenitor responses. To conclude, choosing an optimum dose of statins, based on the mode of drug delivery and the carrier employed, may enhance the positive impact of statins on the periodontal treatment outcomes. Moreover, combining statins with growth factors or other drugs in an efficient carrier system may be beneficial to promote periodontal regeneration.

## Figures and Tables

**Table 1:** Physical properties of different types of statins.

Drug	Source	Solubility	Molecular mass (Da)
Atorvastatin	Synthetic	Lipophilic	1209.42
Simvastatin	Natural	Lipophilic	418.6
Lovastatin	Natural	Lipophilic	404.5
Mevastatin	Natural	Lipophilic	390.52
Pravastatin	Natural	Hydrophilic	446.52
Fluvastatin	Synthetic	Lipophilic	411.47
Cerivastatin	Synthetic	Lipophilic	459.56
Pitavastatin	Synthetic	Lipophilic	421.46
Rosuvastatin	Synthetic	Hydrophilic	481.54

**Table 2:** Representative *in vitro* studies evaluating the impact of statins on periodontal pathogens.

Local drug delivery				
Reference	Experimental design	Type of statin dose	Results	Periodontal consideration
[82]	MIC was determined against *P.g* (ATCC 33277) and *A.a* (ATCC 25586) using serial dilution method	Simvastatin, 1 *μ*g/mL to 500 *μ*g/mL	↘ *P.g* ↘ *A.a*	Simvastatin had an antibacterial effect against the keystone pathogens involved in periodontal disease

[138]	*A.a* (ATCC 43719), *P*. *nigrescens* (ATCC 33563), or *P.g* (ATCC 33277) were cultured on a trilayer functional CS membrane with EGCG and lovastatin	Lovastatin 0.1, 0.5, 1, and 2 mg	↘ *P.g* ↘ *A.a*	Lovastatin had an antibacterial effect against periodontopathogenic bacteria

**Table 3:** *In vivo* studies evaluating the impact of local statin administration on periodontal wound healing.

Local drug delivery				
Reference	Experimental periodontitis induction model (i) Animal (ii) Method (iii) Site	Periodontitis treatment (i) Type of treatment (ii) Type and dose of statin (iii) Mode and time of statin delivery	Results	Periodontal considerations
[139]	Rats (retired female breeder) EIP by ligatures Maxillary right M2	Nonsurgical treatment (therapeutic) Simvastatin prodrug 0.5 mg, 1.0 mg, and 1.5 mg Local injections of the drug/SIM/SIM-mPEG carrier 10 *μ*L into the palatal gingiva between maxillary M1 and M2 Three weekly injections until euthanasia	↗ amount of uninflamed connective tissue in the M1-M2 interproximal area ↘ bone loss, especially with 1.5 mg SIM/SIM-mPEG ↘ percentage of neutrophils	Simvastatin limited periodontal breakdown by reducing bone loss and the extent of gingival inflammation

[73]	Rats (male) ACP (maxillary bone defect) Maxillary M1 extraction followed by socket healing, preparation of a critical-sized periodontal defect (2.0 mm diameter and 1.0 mm depth) on the mesial aspect of the M2, and manual removal of the residual bone and cementum on mesial aspect of M2	Surgical treatment (therapeutic) Simvastatin 1 mg Encapsulated in double-walled PDLLA-PLGA microspheres Combinations: simvastatin-BSA, simvastatin-PDGF, simvastatin	↗ neo-osteogenesis ↗ bone mineral density ↗ bone volume fraction ↗ number and thickness of trabeculae ↘ trabecular separation ↗ cementogenesis of the periodontal apparatus ↘ inflammatory cell infiltration	Simvastatin promoted osteogenic differentiation, reduced inflammation, and facilitated osteogenesis. Sequential PDGF-simvastatin delivery was able to accelerate osteogenesis, bone maturation, fiber realignment, and cementogenesis of the periodontal apparatus, thus accelerating periodontal regeneration

[94]	Rats (male) ACP (tooth-associated alveolar bone defect model) extraction of M1 followed by 4 weeks of socket healing, preparation of a critical-sized intrabony periodontal defect in the M1 edentulous ridge next to the mesial aspect of the M2 finished by a 2.6 mm diameter and 1.0 mm deep osteotomy (completely removing the mesial wall of the osteotomy), and cementum removal (to expose the mesial aspect of M2)	Surgical treatment (therapeutic) Simvastatin 1 mg PDLLA-PLGA hybrid microspheres encapsulating simvastatin/PDGF/BSA to fill the defects	↗ neo-osteogenesis (histologically) PDL fibers not inserted on the root surface (mainly parallel) ↗ bone volume fraction % (not significant)	Simvastatin histologically improved bone healing but better healing response was observed in the group receiving PDGF

[95]	Rats (female) ACP (fenestration defects) Defects 2 mm high, 4 mm wide, and 1.5 mm deep over mandibular molar roots	Nonsurgical treatment (therapeutic) Simvastatin 0.5 mg Local injection of 0.5 mg SIM per site dissolved in 70% ethanol or as SIM-ALN-CD Three weekly injections Treatment started 15 days after the defect preparation	↗ insignificant improvement of bone fill compared to other groups New cementum formation (not significant) But better bone healing response after systemic ALN administration followed by simvastatin injections	Simvastatin had a local bone healing effect which can be augmented by addition of certain other regenerative molecules like ALN

[138]	Dogs (male) ACP (maxillary bone defect) Extraction of all maxillary PM2 followed by healing and preparation of one-walled intrabony defects (4 × 5 × 4 mm: buccolingual, mesiodistal, and depth, respectively) on the mesial and distal sides of maxillary bilateral PM1 Removal of residual cementum by SRP	Surgical treatment (therapeutic) Lovastatin 0.1, 0.5, 1, or 2 mg per trilayer functional CS with the EGCG membrane area (cm^2^)	↗ new bone formation in the EGCG14-CS-lovastatin 1 group (62.03%) > BioMend® group (46.07%) > control group (42.32%) Evidence of new cementum deposition observed on the root surface No inflammatory cell infiltrate was noted in the EGCG_14_-CS-lovastatin 1 group Fibrous connective tissue approximated to the surgical defect	The trilayer functional CS membrane with EGCG and lovastatin enhanced periodontal regeneration and bone formation rate

[140]	Dogs (male) ACP (maxillary bone defect) Extraction of maxillary 2^nd^ and 3^rd^ incisors followed by 8 weeks of socket healing and, later, preparation of three-walled intrabony defects (4 × 4 × 5 mm: buccolingual, mesiodistal, and depth, respectively) on the mesial side of maxillary bilateral canines Removal of residual cementum by SRP	Nonsurgical treatment (therapeutic) Lovastatin 4 mg dissolved in chloroform to form a 3 wt % PLGA solution Local injections of PLGA-lovastatin-CS-tetracycline 0.3% nanoparticles prepared as a hydrogel by mixing with gelatin (10 mg/100 mm^3^) to fill the defects	↗ new deposits of cementum on the root surface ↗ active plasmacytoid osteoblastic rimming along the trabecular surface of the bone adjacent to the defect ↗ percentage of new bone formation (41.32%) No evident inflammation	PLGA-lovastatin-chitosan-tetracycline nanoparticles showed a good osteogenic potential. They promoted new bone and cementum formation

[96]	Rats (male) ACP (mandibular bone defect) Preparation of surgical defects 0.8 mm in diameter through the alveolar bone over the mesiobuccal root of the mandibular M1 bilaterally	Surgical treatment (therapeutic) Simvastatin 2.5% gel Defect was filled with 2.5% simvastatin gel Single topical application	↘ marrow spaces in simvastatin-treated defects ↗ collagen fibril organization ↗ OPN in bone matrix ↗ alveolar bone regeneration	Simvastatin gel improved the quality of the new bone and decreased bone resorption

[99]	Dogs (males and females) ACP (mandibular bone defect) Preparation of bilateral 3-walled intrabony defects (4 × 4 × 4 mm) distal of the mandibular PM2 and mesial of the PM4 and class II furcation defects at the buccal furcation of the mandibular M1 measuring 4 mm occlusal apically and 4 mm buccolingually followed by healing and SRP of defect sites	Nonsurgical treatment (therapeutic) Simvastatin 0.5 mg or 2.0 mg in 30 *μ*L methylcellulose gel Three weekly injections	↗ edentulous ridge thickness (29% greater with simvastatin) ↗ bone loss in class II furcation defects ↗ length of new cementum in the interproximal intrabony defect ↗ bone height with simvastatin (2 mg) No new cementum was observed in furcations	Simvastatin was not appropriate for the treatment of class II furcation defects. However, it improved bone healing in intrabony defects and edentulous ridges significantly

[22]	Rats (male) EIP by ligatures Maxillary M2 bilaterally	Nonsurgical treatment (therapeutic) Atorvastatin 2% *w*/*v* containing CS gel Local 100 *μ*L volume application every other day until euthanasia	↘ IL-1*β*, IL-6, and IL-8 ↗ IL-10 (time dependent) ↘ alveolar bone resorption (significantly with ATV + CS application and insignificantly with ATV alone) ↘ attachment loss Improvement of inflammatory and osteoclastic activity score over time	Atorvastatin with chitosan downregulated inflammation-mediated bone resorption

[90]	Rats (female) EIP by injection of *E. coli* LPS 10 *μ*L of endotoxin injection (1 mg/mL of LPS in PBS) between M1 and M2	Nonsurgical treatment (preventative) Simvastatin 0.5 mg of simvastatin and 3.75 mg of SIM-ALN-CD in H2O Three weekly 12 *μ*L injection bilaterally into the palatal/interproximal gingiva of M1 and M2 Treatment started one week before induction	↗ bone preservation during experimental periodontitis by prophylactic SIM-ALN-CD injection ↘ subsulcular inflammation ↘ alveolar bone loss ↘ OC number	Simvastatin protected against alveolar bone loss and soft tissue inflammation

[98]	Dogs (female) ACP (mandibular bone defect) Preparation of dehiscence defects (5 × 3 mm) bilaterally on the lateral aspect of the mandibular PM2 mesial roots and removal of root cementum Split-mouth design	Surgical treatment (therapeutic) Simvastatin Graft surgery with HA grafts bilaterally covered with resorbable bilayer collagen membranes hydrated with 10 mg simvastatin (graft surgery performed at the time of defect preparation) Local injection 10 mg SIM (0.5 mg/kg) in ethanol (100 *μ*L) Three weekly injections (one week after the graft surgery and defect preparation)	↗ width of new bone in edentulous ridge Distance between CEJ and the alveolar crest was more coronal in dehiscence defects treated with simvastatin (insignificant) Three weeks post-op after simvastatin injection (firm swelling about 1 × 1 cm to 3.5 × 3.5 cm in size), disappeared in 2 months	Simvastatin improved new bone formation where periosteum existed and did not induce severe side effects except for moderate swelling that, eventually, subsided

[59]	Rats (male) EIP by ligatures Left mandibular M1	Nonsurgical treatment (therapeutic) Simvastatin 1 mg/mL (Natrosol + simvastatin gel solution) into the periodontal pocket SRP and irrigation with simvastatin Single injection	↘ MMP-8 expression ↘ bone loss	Simvastatin reduced periodontal bone loss

[141]	Rats (male) EIP by ligatures Maxillary M2	Nonsurgical treatment (therapeutic) Simvastatin 0.2 mg in 50 *μ*L PBS topically injected into the buccal gingivae Twice a week for 70 days	↗ ALP activity ↗ bone nodule formation No inflammatory cells around the new bone ↘ bone loss Simvastatin recovered the ligature-induced alveolar bone resorption (46% reversal of bone height)	Simvastatin increased bone regeneration and reduced inflammation

[142]	Rats (male) EIP by ligatures Mandibular left M1	Nonsurgical treatment (preventative) Simvastatin 0.5 mg/kg body weight orally Followed by laser therapy Treatment started 1 day before induction and daily until euthanasia	↘ bone loss ↘ carbonylated proteins in gingiva	Simvastatin reduced bone loss

[91]	Rats (female ovarectomized) EIP by ligatures Mandibular right M1	Nonsurgical treatment (protective) Simvastatin 10^−6^ M, 3 × 10^−7^ M, 10^−7^ M subperiosteal injections (0.05 mL) Twice a week since the first day of ligature insertion to the 25th day	↘ periodontal breakdown ↘ bone loss in alveolar bone crest zone in a dose-dependent manner (10^−7^ > 10^−6^ > 3 × 10^−7^)	Simvastatin reduced bone loss in a dose-dependent manner

[143]	Rat (female) EIP (ligature) Maxillary M2 bilaterally	Nonsurgical treatment (therapeutic) Simvastatin SIM-PPi conjugate Different treatments including SIM-PPi (dissolved in 25%, 2.56 mg, equivalent to 1.5 mg SIM) and SIM acid (dissolved in PBS, 1.56 mg, equivalent to 1.5 mg of SIM) locally injected (10 *μ*L) into the palatal gingiva between the maxillary M1 and M2 On the first day of weeks 1, 2 and 3 after ligature placement	↗ alveolar bone crest preservation with SIM-PPi ↗ bone volume ↗ trabecular thickness ↗ trabecular number ↘ trabecular separation ↘ neutrophil and lymphocyte score ↘ OC score	Simvastatin improved periodontal bone regeneration and decreased periodontal inflammation

**Table 4:** *In vivo* studies evaluating the impact of systemic statin administration on periodontal wound healing.

Systemic drug delivery				
Reference	Experimental periodontitis induction model (i) Animal (ii) Method (iii) Site	Periodontitis treatment (i) Type of treatment (ii) Type and dose of statin (iii) Mode and time of statin delivery	Results	Periodontal considerations
[31]	Rats (male) EIP by ligatures Maxillary left M2	Nonsurgical treatment (protective) Atorvastatin 1 mg/kg, 5 mg/kg, and 10 mg/kg 1 hour before induction and thereafter once daily	↘ MMP-2, MMP-9 ↘ RANK-L, RANK ↗ OPG ↗ GSH levels ↘ IL-1*β*, TNF-*α*, and MPO (dose dependent) ↘ COX-2 level ↘ MDA activity ↘ alveolar bone loss is dose dependent	Atorvastatin protected against alveolar bone loss in a dose-dependent manner

[58]	Rats (female) EIP by ligatures Maxillary left M2	Nonsurgical treatment (protective) Simvastatin 3, 10, and 30 mg/kg/day 1 hour before induction and thereafter once daily	↗ BMP-2 and OPG levels ↗ TRAP activity ↘ MPO activity (dose dependent) ↘ IL-1*β* and TNF-*α* ↗ IL-10 ↘ gingival GSH ↗ gingival MDA and NOX ↘ iNOS, MMP-1, MMP-8, RANK, and RANKL expression No differences in AST and ALT levels Inhibition of alveolar bone loss	Simvastatin prevented inflammatory bone resorption and possessed antioxidant properties

[144]	Rats (male) EIP by ligatures Maxillary left M2	Nonsurgical treatment (protective) Atorvastatin 1, 3, and 9 mg/kg Atorvastatin mixed in sterile saline by gavage 30 min before ligature placement and then daily until euthanasia	↘ alveolar bone loss in the furcation area as well as in proximal faces of upper M2 (47% reduction with 9 mg dose compared to that with the control) Insignificant bone loss protection with 1 and 3 mg doses	Atorvastatin had protective effect against alveolar bone loss

[40]	Rats (male) EIP by ligatures Maxillary left M2	Nonsurgical treatment (protective + therapeutic) Atorvastatin 0.3 mg/kg or 27 mg/kg by gavage In combination with ALN 30 min before ligature placement and thereafter once daily until euthanasia or 5 days after the start of periodontitis induction and then daily until euthanasia	↘ TRAP and MPO activity ↘ cementum resorption ↘ neutrophilia and lymphomonocytosis ↘ alveolar bone loss both prophylactically (39%) and therapeutically (53.4%) with lower dose of ALN + ATV (0.01 mg/kg+0.3 mg/kg, respectively) Prevented BALP reduction with lower dose of ALN + ATV No effect on serum transaminases	Atorvastatin reduced alveolar bone loss, cemental resorption, and inflammatory cell infiltration both prophylactically and therapeutically

[145]	Rats (male) EIP by ligatures Maxillary left M2	Nonsurgical treatment (protective) Atorvastatin 0.3, 3, and 27 mg/kg by gavage 30 min before ligature placement and thereafter once daily until euthanasia	↘ alveolar bone in a dose-dependent manner (39% for 3 mg/kg and 56% for 27 mg/kg doses) Prevented the reduction of BALP serum levels (27 mg/kg) Prevented leukocytosis (27 mg/kg)	Atorvastatin prevented alveolar bone loss with both prophylactic and therapeutic doses

[32]	Rats (female with metabolic syndrome) ACP (injection of 20 *μ*g of *A.a* LPS in PBS) into the palatal gingiva between the maxillary M1 and M2, thrice per week for 4 weeks	Nonsurgical treatment (protective) Simvastatin 20 mg/kg/day Daily via gavage for 4 weeks Treatment started on the same day as injection of LPS	↘ LPS induced alveolar bone loss in both lean and fat rats (significantly) ↘ infiltration of mononuclear cells ↘ inflammatory score ↘ LPS stimulated RANKL and CSF2 expression in both lean and fat rats ↘ bone resorption	Simvastatin downregulated inflammation-mediated bone resorption

[33]	Rats (female) *ACP injection of* 20 *μ*g/rat of *A.a* LPS through the palatal gingiva between the maxillary M1 and M2 thrice per week for 8 weeks	Nonsurgical treatment (protective) Simvastatin (20 mg/kg/day) daily via oral gavage for 8 weeks	↘ LPS induced alveolar bone loss (31%) ↘ LPS induced osteoclastogenesis ↘ TNF-*α*, IL-1*α*, IL-1*β*, IL-6, CSF-2, CSF-3, MCP-1, and MMP-9 ↘ LPS induced TLR family members' expression	Simvastatin downregulated inflammation-mediated bone resorption

[25]	Rats (male) EIP by ligatures Maxillary M2	Nonsurgical treatment (protective) Rosuvastatin 20 mg/kg in water by gavage 1 h before ligation and then once daily until euthanasia	↗ IL-10 ↘ IL-1*β* ↗ MDA ↗ GSH ↘ inflammatory infiltrate ↘ OC number ↗ OB number ↘ alveolar bone loss (significantly)	Rosuvastatin protected against alveolar bone loss

[54]	Rats (male) EIP by ligatures Hyperlipidemia induction through diet Maxillary M2	Nonsurgical treatment (protective) Rosuvastatin 20 mg/kg in water by gavage 1 h before ligation and then once daily until euthanasia	↘ gingival iNOS (significantly) ↘ inflammation and hyperemia ↘ alveolar bone loss	Rosuvastatin protected against inflammation-induced bone degradation

[34]	Rats (male) EIP by ligatures Mandibular M1 and maxillary M2 bilaterally	Nonsurgical treatment (therapeutic) Simvastatin 10 mg/kg in water once daily orally until euthanasia Treatment started 8 days after periodontitis induction	↘ alveolar bone loss ↘ IL-6 ↘ CRP	Simvastatin decreased inflammation and alveolar bone loss

[93]	Rats (male hypertensive) EIP by ligatures Mandibular M1 bilaterally	Nonsurgical treatment (protective) Rosuvastatin 2 mg/kg oral gavage Treatment started since the day of induction daily until euthanasia	↘ bone loss in furcation area ↘ attachment loss ↘ TRAP-positive multinucleated cells	Rosuvastatin reduced alveolar bone loss and osteoclastogenesis

[97]	Rats EIP by ligatures Mandibular M1	Nonsurgical treatment (protective + therapeutic) Simvastatin Different treatments: simvastatin-simvastatin: aqueous suspension of simvastatin by gavage (35 mg/kg/day) administration before and after periodontitis induction; simvastatin-water: simvastatin administration before and filtered water after periodontitis induction; and water-simvastatin: water administration before and simvastatin after periodontitis induction	No significant differences between groups receiving simvastatin before the induction of periodontitis and those that received water No protective effect of simvastatin against the development of periodontitis	Simvastatin did not possess protective or therapeutic effects against periodontitis development

[146]	Rats (male) EIP by ligatures Mandibular left M1	Nonsurgical treatment (therapeutic) Simvastatin 25 mg/kg Dissolved in saline Treatment started 14 days after the initiation of periodontitis induction	↗ TG levels ↘ MDA level ↗ IL-10 ↘ MMP-9 ↘ bone loss No difference on TNF-*α* levels	Simvastatin promoted the anti-inflammatory mediators to counter alveolar bone loss

[35]	Rats (male, cyclosporine A-induced alveolar bone loss) EIP by ligatures Mandibular right M1	Nonsurgical treatment (protective) Simvastatin 20 mg/kg orally daily for 30 days The treatment and induction started on the same day	↗ Ca2+ concentrations (significantly) No effect of simvastatin treatment in the presence of periodontal disease on serum ALP levels but it blocked the cyclosporine A-mediated decrease of ALP No significant effect on alveolar bone turnover but with concomitant cyclosporine A and simvastatin delivery Simvastatin completely inhibited cyclosporine A-induced bone loss	Simvastatin did not prevent alveolar bone loss in periodontitis but it completely countered the cyclosporine A-induced bone loss

[147]	Rats (male) EIP by ligatures Mandibular right M1	Nonsurgical treatment (protective) Simvastatin 20 mg/kg The treatment and induction started on the same day	↗ ALP activity in periodontal inflammation ↘ alveolar bone loss	Simvastatin protected against alveolar bone loss

[76]	Mice (male) ACP (*P.g* LPS injection) 1 mg/kg *P.g* LPS injection at the gingiva of left mandibular M2 on days 4 and 7	Nonsurgical treatment (protective) Fluvastatin 3 mg/kg IP injections on days 1, 4, and 7	↘ LPS induced OC (by >50%) ↘ LPS-induced bone erosion ↘ RANKL	Fluvastatin prevented inflammation-induced bone erosion

[26]	Rats (male, GIOP) EIP by ligatures Maxillary left M2	Nonsurgical treatment (protective) Atorvastatin 27 mg/kg ATV orally 30 min before induction and once daily afterwards	↘ bone loss ↘ MPO, TNF-*α*, IL-1*β*, IL-6, and IL-8 ↗ IL-10, GSH, SOD, and CAT levels ↘ RANKL and DKK-1 ↗ OPG, WNT10 *β*, and *β*-catenin expressions and BALP activity	Atorvastatin prevented alveolar bone loss in periodontitis and reduced inflammation

**Table 5:** *In vivo* studies evaluating the impact of a combination of local and systemic statin administration on periodontal wound healing.

Local + systemic drug delivery				
Reference	*Experimental periodontitis induction model* (i) Animal (ii) Method (iii) Site	*Periodontitis treatment* (i) Type of treatment (ii) Type and dose of statin (iii) Mode and time of statin delivery	Results	Periodontal considerations
[57]	Rats (male) EIP by ligature mandibular M1	Nonsurgical treatment (therapeutic) Atorvastatin Systemically (5 mg/kg in a volume of 0.5 mL) and locally (0.1 mg/kg in a volume of 0.05 mL) at a dose of 0.1 mg/kg in a volume of 0.05 mL	↗ alveolar bone area % ↗ VEGF ↘ MMP-9 ↘ alveolar bone and attachment loss Local application showed better results on periodontium healing	Atorvastatin increased the alveolar bone regeneration while decreasing the periodontal inflammation and attachment loss

[92]	Rats (female ovarectomized) EIP by ligatures Maxillary M1 and M2 bilaterally	Nonsurgical treatment (therapeutic) Simvastatin Local injection (0.8 mg/0.05 mL) Oral (25 mg/kg) For two months until euthanasia	↗ alveolar crest height (28% with local & oral and 27% with local) ↗ BV/TV ↗ trabecular thickness ↘ trabecular separation	Simvastatin reduced bone degradation when administered locally, systemically, or both locally and systemically together

The animals included in the studies are healthy unless stated otherwise. Treatment was considered (1) “preventative” when it started at least one day before the start of EIP/ACP induction, (ii) “protective” when it started the same day as that of EIP/ACP induction, and (iii) “therapeutic” when it started at least one day after the start of EIP/ACP induction.

**Table 6:** Clinical studies evaluating the impact of local statin administration on periodontal wound healing.

Local drug delivery					
Reference Study area Type of study	Drug Mode of delivery Dose	Number of patients Periodontal status Type of patients	Type of treatment Study design (groups) Follow-up	Results	Periodontal considerations
[130] (India) RCT with split-mouth design	Simvastatin in methylcellulose gel 1.2 g of SIM	30 Periodontitis (Armitage 1999) Healthy patients (nonsmokers) Sites with periodontal pocket measuring ≥ 5 mm and vertical bone loss ≥ 2 mm in different quadrants of the mouth	Nonsurgical treatment Group I: SRP + placebo gel Group II: SRP + SIM gel 6 months follow-up	All subjects tolerated the drug ↗ periodontal parameters with or without SIM ↗ CAL (*p* = 0.02) ↗ INFRA 2 (*p* < 0.01) ↘ PD significantly (*p* = 0.04) ↘ INFRA 1 (*p* < 0.01)	Simvastatin increased periodontal regeneration and CAL gain

[23] (India) RCT	Rosuvastatin 1.2% rosuvastatin (RSV) gel	90 Chronic periodontitis Healthy patients (nonsmokers)	Nonsurgical treatment Groups I: SRP + placebo gel Group II: SRP + 1.2% RSV gel Group III: SRP + 1% MF gel 12 months follow-up	↗ CAL ↘ PD significant ↗ bone fill ↘ PI ↘ mSBI ↘ DDR	Rosuvastatin increased periodontal regeneration and CAL gain

[102] (India) RCT	Atorvastatin and rosuvastatin 1.2% atorvastatin or 1.2% rosuvastatin gel local drug delivery (1.2 mg/0.1 mL)	90 No data Healthy patients (nonsmokers) Mandibular class II furcation defects with PD ≥ 5 mm and horizontal PD ≥ 3 mm	Nonsurgical treatment Group I: SRP + placebo Group II: SRP + 1.2% RSV gel Group III: SRP + 1.2% ATV gel 9 month follow-up	↘ PI and mSBI in all groups The 2 statins lead to the following: ↘ PD ↗ mean gain in CAL ↗ mean percentage of DDR Statistically greater results for RSV than for ATV	Statins increased periodontal regeneration and CAL gain

[103] (India) Cohort study	Simvastatin SIM gel (1.2 mg/0.1 mL)	50 Chronic periodontitis Healthy patients (nonsmokers)	Nonsurgical treatment Group I: SRP alone Group II: SRP + SIM gel 3 months follow-up	↘ IL-6 and IL-8 ↗ IL-10 significantly ↘ PI, mSBI, and PD No effect on CAL	Simvastatin gel decreased periodontal inflammation and promote periodontal regeneration

[21] (India) RCT	Simvastatin 1.2% simvastatin gel	46 Chronic periodontitis Healthy patients (nonsmokers)	Nonsurgical treatment Group I: SRP Group II: SRP + SIM gel 45 days follow-up	↘ PI, GI, and SBI No significant difference for PD and CAL ↘ mean IL-6 levels No significant difference for IL-8 levels	Simvastatin gel decreased periodontal inflammation

[104] (India) Cohort study with split-mouth design	Simvastatin Combination of DFDBA and a 10^−8^ M solution of the drug simvastatin	15 No data Healthy patients (nonsmokers) Identical bilateral infrabony defect	Surgical treatment (Kirkland flap) Group A: DFDBA alone Group B: DFDBA + SIM 24 weeks follow-up	↘ PD ↗ mean gain in CAL (better with DFDBA + SIM) ↘ infrabony defect depth (greater reduction with DFDBA + SIM) ↗ linear defect fill (better with DFDBA + SIM)	Simvastatin increased periodontal regeneration and CAL gain

[148] (India) RCT	Atorvastatin 1.2% atorvastatin gel (ATV gel (1.2 mg/0.1 mL)	75 Well-controlled type 2 diabetic patients (nonsmokers) Chronic periodontitis	Nonsurgical treatment Group 1: SRP + ATV Group 2: SRP + placebo 9 months follow-up	↗ mSBI ↘ PD ↗ CAL gain ↘ IBD depth and DDR No significant difference for PI at all time intervals evaluated	Atorvastatin increased periodontal regeneration

[125] (India) RCT	Atorvastatin 1.2% atorvastatin gel (ATV gel (1.2 mg/0.1 mL))	71 Smokers Chronic periodontitis	Nonsurgical treatment Group 1: SRP + ATV Group 2: SRP + placebo 9 months follow-up	↘ PD ↗ mean CAL gain ↘ mean percentage of DDR ↘ mSBI ↘ IBD depth No statistically significant difference in the site-specific PI score and full-mouth PI score between the groups at any visit	Atorvastatin increased periodontal regeneration and CAL gain

[105] (India) Cohort	Atorvastatin 1.2% ATV gel	96 Healthy patients (nonsmokers) Chronic periodontitis	Surgical treatment Group I: OFD + PRF Group II: OFD + PRF + 1.2% ATV Group III: OFD alone 9 months follow-up	ATV gel and PRF alone showed significantly the following: ↘ PD ↗ mean CAL gain ↘ IBD depth No statistically significant difference in PI and mSBI scores between the groups at 9 months	Atorvastatin increased periodontal regeneration and CAL gain

[101] (India) RCT	Atorvastatin and simvastatin 10 mL of 1.2% ATV gel (1.2 mg/0.1 mL) and 10 mL of 1.2% SIM gel (1.2 mg/0.1 mL)	96 Healthy patients (nonsmokers) Chronic periodontitis	Nonsurgical treatment Group I: SRP + 1.2% ATV Group II: SRP + 1.2% SIM Group III: SRP + placebo 9 months follow-up	The 2 statins lead to the following: ↘ PD ↘ mSBI ↘ IBD depth ↗ mean CAL gain Statistically greater results for ATV than for SIM for PD reduction, CAL gain and percentage of IBD reduction	Atorvastatin increased periodontal regeneration and CAL gain

[149] (India) RCT	Simvastatin Single topical transmucosal injection 1.2 mg SIM	60 Chronic periodontitis Healthy patients (nonsmokers)	Nonsurgical treatment Group I: SRP + placebo Group II: SRP + SIM 6 months follow-up	↘ mSBI ↘ mean PD ↗ mean CAL ↗ IBD fill ↘ GI	Simvastatin increased periodontal regeneration and CAL gain

[126] (India) RCT	Simvastatin SIM 1.2 *μ*g/inj. (0.12 *μ*g/mm3) Methylcellulose gel	72 Chronic periodontitis Healthy patients (nonsmokers) Mandibular buccal class II furcation defects	Nonsurgical treatment Group I: SRP + placebo Group II: SRP + 1.2 mg SIM 6 months follow-up	↘ SBI and PB ↗ CAL ↗ IBD fill	Simvastatin increased periodontal regeneration and CAL gain

[150] (India) RCT	Atorvastatin 1.2% ATV methyl cellulose gel	60 patients Chronic periodontitis Healthy patients (nonsmokers)	Nonsurgical treatment Group I: SRP + 1.2% ATV Groups II: SRP + placebo gel 9 months follow-up	↘ PD ↘ mSBI ↗ mean CAL gain ↗ IBD fill	Simvastatin increased periodontal regeneration and CAL gain

[151] (India) RCT	Simvastatin 1.2% SIM gel	38 Chronic periodontitis Well-controlled type II diabetes Nonsmokers	Nonsurgical treatment Group I: SRP + SIM Group II: SRP + placebo 9 months follow-up	↘ PD ↗ mean CAL gain ↗ mean radiographic bone fill ↘ mSBI	Simvastatin increased periodontal regeneration and CAL gain

[152] (India) RCT	Rosuvastatin 1.2% rosuvastatin (RSV) gel	65 Chronic periodontitis Healthy (nonsmokers)	Nonsurgical treatment Group I: SRP + RSV Group II: SRP + placebo 6 months follow-up	↘ mSBI ↘ PD ↗ mean CAL gain ↗ IBD fill	Rosuvastatin increased periodontal regeneration and CAL gain

[20] (India) RCT	Atorvastatin + rosuvastatin 1.2% RSV and 1.2% ATV gel	90 Chronic periodontitis Healthy (nonsmokers)	Nonsurgical treatment Group I: SRP + placebo Group II: SRP + 1.2% RSV gel Group III: SRP + 1.2% ATV gel 9 months follow-up	The 2 statins lead to the following: ↘ mSBI ↘ PD ↗ mean CAL gain ↗ IBD fill Statistically greater results for RSV than for ATV for PD reduction, CAL gain, IBD reduction, and msSBI reduction	Atorvastatin and rosuvastatin increased periodontal regeneration and CAL gain

[106] (India) RCT	Rosuvastatin 1.2% RSV gel	90 Chronic periodontitis Healthy (nonsmokers)	Surgical treatment 2/3-walled intrabony defects Group I: OFD alone Group II: OFD + PRF Group III: OFD + PRF + 1.2% RSV gel 9 months follow-up	↘ PD ↗ mean CAL gain ↗ IBD fill	Rosuvastatin increased periodontal regeneration and CAL gain

[107] (India) RCT	Rosuvastatin 1.2% RSV gel	110 Chronic periodontitis Healthy (nonsmokers) Mandibular degree II furcation defects	Surgical treatment Group 1: OFD + placebo gel Group II: OFD + PRF + HA Group III: OFD + RSV 1.2 mg gel + PRF + HA 9 months follow-up	↘ PD ↗ mean CAL gain ↗ IBD fill ↘ PI and mSBI	Rosuvastatin increased periodontal regeneration and CAL gain

[153] (India) RCT	Atorvastatin 1.2% atorvastatin gel	90 Chronic periodontitis Healthy patients (nonsmokers) Intrabony defect	Nonsurgical treatment Group I: SRP + ALN Group II: SRP + 1.2% ATV Group III: SRP + placebo group 9 months follow-up	↘ PD ↗ mean CAL gain ↗ IBD fill ↘ mSBI	Local delivery of atorvastatin increased periodontal regeneration

[154] (India) RCT	Simvastatin 0.1 mL SIM gel (1.2 mg/0.1 mL)	24 Aggressive periodontitis Healthy patients (nonsmokers) Intrabony defect	Nonsurgical treatment Group I: SRP + placebo gel Group II: SRP + SIM gel 6 months follow-up	↘ PD ↗ mean CAL gain ↗ IBD fill ↘ mSBI All patients tolerated the drug with no postapplication complications No statistically significant difference between groups I and II regarding PI	Simvastatin increased periodontal regeneration

[108] (India) RCT	Simvastatin 1.2 mg Simvastatin gel	20 Chronic periodontitis Healthy patients (nonsmokers)	Surgical treatment PD ≥ 5 mm in the mandibular molar region bilaterally Group I: OFD + SIM Group II: OFD + placebo gel 9 months follow-up	↗ IBD fill for group I Significant results at 9 months in both groups: ↘ GI, PD ↗ mean CAL gain	Simvastatin increased periodontal regeneration

[155] (India) RCT	Simvastatin 10 *μ*L prepared SIM gel (1.2 mg/0.1 mL)	40 Chronic periodontitis Healthy patients Smokers only	Nonsurgical treatment Group I: SRP + SIM 1.2% Group II: SRP + placebo 9 months follow-up	↘ mSBI ↘ PD ↗ mean CAL gain ↗ IBD fill	Simvastatin increased periodontal regeneration and CAL gain

[156] (India) RCT	Simvastatin 1.2% simvastatin gel	60 Chronic periodontitis Healthy (nonsmokers)	Nonsurgical treatment Group A: SRP + placebo Group B: SRP + SIM gel 6 months follow-up	↘ mSBI and PD ↗ mean CAL gain ↗ IBD fill ↘ IL-6 levels	This study showed the efficacy of SIM as a local drug delivery system in the treatment of chronic periodontitis not only in clinical but also in molecular levels

[137] (Chile) RCT	Atorvastatin 2% atorvastatin dentifrice	36 Chronic periodontitis Controlled diabetic only All types of smoking status	Nonsurgical treatment Group I: SRP + ATV dentifrice Group II: SRP + placebo dentifrice 1 month follow-up	↘ PISA ↘ mean PD ↘ % of sites with PD ≥ 5 mm ↗ mean CAL gain ↘ % of sites with CAL ≥ 5 mm ↘ BOP ↘ GI	Simvastatin increased periodontal regeneration and CAL gain

[100] (India) Cohort study	Atorvastatin + simvastatin Drug in sodium alginate suspension administered with calcium chloride solution, subgingival delivery 1.2% simvastatin, or 1.2% atorvastatin	45 Moderate to severe chronic periodontitis Healthy (nonsmokers)	Nonsurgical treatment Group I: SRP alone Group II: SRP + 1.2% SIM Group III: SRP + 1.2% ATV 6 months follow-up	The test groups did not show any statistically significant difference when compared with the control group	No significant benefit for periodontal regeneration with the use of statin

**Table 7:** Clinical studies evaluating impact of systemic statin administration on periodontal wound healing.

Systemic drug delivery					
Reference Study area Type of study	Drug Mode of delivery Dose	Number of patients Periodontal status Type of patients	Type of treatment Study design (groups) Follow-up	Results	Periodontal considerations
[109] (USA) Retrospective cohort study	Not reported	1021 Chronic periodontal disease All types of patients (diabetic, smokers, antibiotic users, anti-inflammatory users…)	Nonsurgical treatment Hyperlipidemic vs healthy Mean follow-up = 7.1 years	Any statin use during the first 3 years after the initial periodontal exam was associated with a 48% decreased tooth loss rate in year 4 and subsequent years	Statins reduced tooth loss in chronic periodontitis

[112] (Mexico) RCT	Atorvastatin 20 mg/day	38 Chronic periodontitis Healthy (all types of smoking status)	Nonsurgical treatment Group I: SRP + ATV Group II: SRP + placebo 3 months follow-up	↘ dental mobility ↘ distance from the crestal alveolar bone to the cementoenamel junction	Atorvastatin reduced tooth mobility and bone loss

[110] (Turkey) No control group Longitudinal	Atorvastatin 10 or 20 mg	20 Chronic periodontitis Hyperlipidemic patients (nonsmokers)	Nonsurgical treatment SRP 6 months follow-up	↘ median values for the PI, GI, PD, and BOP (%) ↗ median value of CAL gain All lipid parameters decreased after the periodontal treatment No comparison with the control group	Atorvastatin reduced periodontal breakdown Improved periodontal health may influence metabolic control of hyperlipidemia

[113] (Turkey) Cohort study	Atorvastatin 10 or 20 mg	80 Chronic periodontitis Healthy or hyperlipidemic patients (nonsmokers)	Nonsurgical treatment Group I: healthy patient + SRP Group II: hyperlipidemic patients + prescribed diet (HD) Group III: hyperlipidemic patients + atorvastatin (HS) 3 months follow-up	↗ BOP ↘ IL-6 (serum and GCF) ↘ TNF-*α* (GCF) levels	Systemic atorvastatin had beneficial effects on periodontal inflammation

[111] (Germany) Cohort study	Simvastatin (*n* = 87), lovastatin (*n* = 27), pravastatin (*n* = 53), fluvastatin (*n* = 37), atorvastatin (*n* = 34), and cerivastatin (*n* = 42)	2689 All types of periodontal disease Hyperlipidemic vs normolipidemic All types of smoking status	All types of periodontal treatment Group I: participants undergoing statin treatment Group II: patients without statins 5.3 years mean follow-up	No effect on PD and CAL ↘ tooth loss	Statins had the beneficial effect of protecting against tooth loss

[56] (USA) Cohort study	Simvastatin Not reported	117 Chronic periodontitis Diabetic vs healthy All types of smoking status	Nonsurgical treatment Group I: nondiabetic patients not taking statin Group II: nondiabetic patients taking statin Group III: diabetic patients not taking statin Group IV: diabetic patients taking statin 6 weeks follow-up	↘ PD in diabetic patients ↗ CAL in nondiabetic patients ↘ MMP-1 level in GCF of nondiabetic and diabetic patients No difference was found for MMP-8 and MMP-9 levels in GCF	Statin intake was associated with reduced PD in diabetic patients and MMP-1 level in GCF in either nondiabetic or diabetic patients

[114] (India) Cohort study	Atorvastatin 20 mg/day	107 Chronic periodontitis Hyperlipidemic vs normolipidemic Nonsmokers	Nonsurgical periodontal treatment Group 1: hyperlipidemic + SIM Group 2: hyperlipidemic + diet Group 3: normolipidemic patients 3 months follow-up	↘ GI Mean change in PD is negatively associated with LDL-C Mean change in GI is positively associated with HDL-C	Patients with hyperlipidemia were more prone to periodontal disease Statin intake had beneficial effects on periodontal inflammation

## Abbreviations

M1: First molar
M2: Second molar
M3: Third molar
mPEG: Polyethylene glycol monomethyl ether
PDLLA-PLGA: Poly-(d,l-lactide) and poly-(d,l-lactide-co-glycolide
BSA: Bovine serum albumin
PDGF: Platelet-derived growth factor
PM: Premolar
PDL: Periodontal ligament cells
EGCG: Epigallocatechin-3-gallate
CS: Chitosan
BALP: Bone alkaline phosphatase
LPS: Lipopolysaccharide
PBS: Phosphate buffered saline
ALN-CD: Alendronate-*β*-cyclodextrin
SIM: Simvastatin
CEJ: Cementoenamel junction
HA: Hydroxyapatite
TGF-*β*: Transforming growth factor beta
*E. coli*: *Escherichia coli*
PPi: Isopropyl alcohol
TRAP: Tartrate-resistant acid phosphatase
GSH: Glutathione
MDA: Malondialdehyde
MPO: Myeloperoxidase
GIOP: Glucocorticoid-induced osteoporosis
DKK1: Dickkopf-related protein
CAT: Enzyme catalase
SOD: Enzyme superoxide dismutase
MMPs: Matrix metalloproteinases
MCP: Monocyte chemotactic protein
CSF: Colony-stimulating factor
*A*.*a*: *Aggregator actinomycetemcomitans*
*P.g*: *Porphyromonas gingivalis*
COX: Cyclooxygenase
ALP: Alkaline phosphatase
AST: Aspartate aminotransferase
ALT: Alanine aminotransferase
IP: Intraperitoneal
TG: Triglyceride
ATV: Atorvastatin
PD: Pocket depth
RANKL: Receptor activator of the NF-*κ*B ligand
RANK: Receptor activator of NF-*κ*B
OPG: Osteoprotegerin
OPN: Osteopontin
BV/TV: Bone volume/tissue volume
CAL: Clinical attachment level
SRP: Scaling and root planing
INFRA: Radiographic infrabony defect fill
MF: Metformin
DDR: Defect depth reduction
DFDBA: Demineralized freeze-dried bone allograft
OFD: Open flap debridement
BOP: Bleeding on probing
GI: Gingival index
PI: Plaque index
mSBI: Modified sulcus bleeding index
IBD: Intrabony defect
PRF: Platelet-rich fibrin
PISA: Periodontal inflamed surface area
LDL-C: Low-density lipoprotein cholesterol
HDL-C: High-density lipoprotein cholesterol
OB: Osteoblasts
OC: Osteoclasts
EIP: Experimentally induced periodontitis
ACP: Acute/chronic periodontal defect
NOX: Nitrate/nitrite levels
VEGF: Vascular endothelial growth factor.

## Supplementary Materials

Supplementary Materials Risk of bias assessment of included clinical studies.
