# Supplementary material for: Contribution of Statins towards Periodontal Treatment: A Review
Source: Mediators Inflamm. 2019 Feb 27;2019:6367402. doi: 10.1155/2019/6367402 (PMC6415285; doi:10.1155/2019/6367402)
Supplement: Supplementary Materials — Risk of bias assessment of included clinical studies. [file 6367402.f1.pdf]

Supplemental table 1: Risk of bias assessment

Study	Random sequence generation	Allocation concealment	Blinding of participants and personnel	Blinding of outcome assessment	Incomplete outcome data	Selective reporting	Other sources of bias
Agarwal et al. 2016	Low risk	High risk	Low risk	Low risk	Low risk	Low risk	Low risk
Cunha-cruz et al. 2006	High risk	High risk	High risk	High risk	High risk	High risk	High risk
Dileep et al. 2018	Low risk	High risk	Low risk	Low risk	Low risk	Low risk	Low risk
Fajardo et al. 2010	Low risk	Low risk	Low risk	Low risk	Low risk	Low risk	Low risk
Fentoğlu et al. 2012	High risk	High risk	High risk	High risk	Low risk	Low risk	High risk
Fentoğlu et al. 2010	High risk	High risk	High risk	High risk	Low risk	Low risk	Low risk
Garg et al. 2017	Low risk	High risk	Low risk	Low risk	Low risk	Low risk	Low risk
Grover et al. 2016	High risk	High risk	High risk	High risk	Low risk	Low risk	Low risk
Gunjiganur et al. 2017	Low risk	High risk	Low risk	Low risk	Low risk	Low risk	Low risk
Kinra et al. 2010	High risk	High risk	Low risk	Low risk	Low risk	Low risk	Low risk
Kumari et al. 2016	Low risk	High risk	Low risk	Low risk	Low risk	Low risk	Low risk
Kumari et al. 2017	Low risk	High risk	Low risk	Low risk	Low risk	Low risk	High risk
Martande et al. 2016	Low risk	High risk	Low risk	High risk	Low risk	Low risk	Low risk
Martande et al. 2017	Low risk	High risk	Low risk	High risk	Low risk	Low risk	High risk
Meisel et al. 2014	High risk	High risk	High risk	High risk	Low risk	Low risk	High risk
Priyanka et al. 2017	Low risk	High risk	Low risk	Low risk	Low risk	Low risk	Low risk
Poston et al. 2016	High risk	High risk	High risk	High risk	Low risk	Low risk	Low risk

Pradeep et al. 2010	Low risk	High risk	Low risk	Low risk	Low risk	Low risk	High risk
Pradeep et al. 2012	Low risk	High risk	Low risk	Low risk	Low risk	Low risk	Low risk
Pradeep et al. 2013	Low risk	High risk	Low risk	Low risk	Low risk	Low risk	Low risk
Pradeep et al. 2013	Low risk	High risk	Low risk	Low risk	Low risk	Low risk	Low risk
Pradeep et al. 2015	Low risk	High risk	Low risk	Low risk	Low risk	Low risk	High risk
Pradeep et al. 2015	Low risk	High risk	Low risk	High risk	Low risk	Low risk	Low risk
Pradeep et al. 2016	Low risk	High risk	Low risk	Low risk	Low risk	Low risk	Low risk
Pradeep et al. 2016	Low risk	High risk	Low risk	Low risk	Low risk	Low risk	Low risk
Pradeep et al. 2017	Low risk	High risk	Low risk	Low risk	Low risk	Low risk	High risk
Rajan et al. 2017	Low risk	High risk	Low risk	Low risk	Low risk	Low risk	Low risk
Rao et al. 2013	Low risk	High risk	Low risk	Low risk	Low risk	Low risk	Low risk
Rath et al., 2012	Low risk	High risk	High risk	High risk	Low risk	Low risk	High risk
Rosenberg et al. 2015	Low risk	Low risk	Low risk	Low risk	Low risk	Low risk	Low risk
Sangwan et al. 2016	High risk	High risk	Low risk	Low risk	Low risk	Low risk	Low risk
Surve et al. 2015	High risk	High risk	High risk	High risk	Low risk	Low risk	Low risk
